# The Mammalian Circadian Clock Gene *Per2* Modulates Cell Death in Response to Oxidative Stress

**DOI:** 10.3389/fneur.2014.00289

**Published:** 2015-01-13

**Authors:** Maria Chiara Magnone, Sonja Langmesser, April Candice Bezdek, Tiziano Tallone, Sandro Rusconi, Urs Albrecht

**Affiliations:** ^1^Department of Biology, Division of Biochemistry, University of Fribourg, Fribourg, Switzerland

**Keywords:** apoptosis, adenovirus, *bcl-2*, paraquat, plumbagin, UV, SIN-1, p53

## Abstract

Living in the earth’s oxygenated environment forced organisms to develop strategies to cope with the damaging effects of molecular oxygen known as reactive oxygen species (ROS). Here, we show that *Per2*, a molecular component of the mammalian circadian clock, is involved in regulating a cell’s response to oxidative stress. Mouse embryonic fibroblasts (MEFs) containing a mutation in the *Per2* gene are more resistant to cytotoxic effects mediated by ROS than wild-type cells, which is paralleled by an altered regulation of *bcl-2* expression in *Per2* mutant MEFs. The elevated survival rate and alteration of NADH/NAD^+^ ratio in the mutant cells is reversed by introduction of the wild-type *Per2* gene. Interestingly, clock synchronized cells display a time dependent sensitivity to paraquat, a ROS inducing agent. Our observations indicate that the circadian clock is involved in regulating the fate of a cell to survive or to die in response to oxidative stress, which could have implications for cancer development and the aging process.

## Introduction

Life on earth is under the continuous influence of a light and dark cycle that is caused by the rotation of the earth around its axis and its orbit around the sun. Many organisms have internalized the cyclic change of light and darkness in the form of the circadian clock. This enables them to predict dusk and dawn giving them an edge in competing for limited resources and avoiding predators (1, 2). The circadian timing system provides a temporal organization within an organism to modulate and synchronize biological functions in order to prevent the activation of biochemical pathways that would counteract each other. During the day, catabolic processes facilitate engagement with the environment, whereas at night, anabolic functions of growth, repair, and consolidation predominate (3).

The mechanism underpinning the circadian clock is found in most cells of the body including fibroblasts (4). It comprises a set of clock genes that are organized in a positive and a negative limb constituting an autoregulatory feedback loop. The positive limb involves CLOCK:BMAL1 heterodimers that bind to E-box promoter elements located in the regulatory region of the *Period* (*Per1*, *Per2*) and *Cryptochrome* (*Cry1*, *Cry2*) genes. Their proteins form oligomers that are transported from the cytoplasm to the nucleus where they repress their own transcription (negative limb) (5). The positive and negative limbs are interlaced by the nuclear orphan receptor REV-ERBα repressing transcription of *Bmal1* through binding to a RORE element present in the *Bmal1* promoter (6). Interestingly, the molecular clock components do not only influence their own transcription but also regulate the expression of a large number of genes belonging to diverse biochemical pathways. Clock regulated key pathways include detoxification, oxidative phosphorylation, and energy metabolism (7, 8). Therefore, one could expect that an organism with a defective circadian clock would have difficulties in coping with environmental stress. This view is supported by the finding that mice mutant in the *Per2* gene [*Per2^Brdm1^*, Ref. (9)] are cancer-prone after γ irradiation (10). The effects of this DNA damaging treatment might also be related to abnormal responses of *Per2^Brdm1^* mutant cells to oxidative stress.

Oxidative stress can be caused by intra- or extracellular sources of reactive oxygen species (ROS), such as O_2_ radicals, which are produced in mitochondria during oxidative phosphorylation or are generated due to genotoxic stress by agents such as paraquat. In a cell, oxidative stress affects the production of low molecular mass antioxidants (e.g., vitamin C, tocopherol, lipoic acid) and the induction as well as the activation of antioxidant enzymes, such as catalase, glutathione peroxidase, and superoxide dismutases (SODs). A disturbance of the balance between pro-oxidant:antioxidant processes leads to alterations in redox homeostasis and oxidation of DNA and cellular biomolecules. In a first line, the cell may respond by autophagy, in order to remove damaged cellular components and damaged organelles. Alternatively, the cell can undergo either necrotic cell death or programed cell death (apoptosis). However, the switch between the different possibilities for a cell to respond to oxidative stress is not completely understood [reviewed in Ref. (11)].

Since the circadian clock integrates and regulates metabolism, we started to investigate whether the circadian clock is involved in cellular response to oxidative stress. In particular, we were interested in the role of the *Per2* gene, which is known to be one of the clock components responding to external signals such as light (12) and temperature (13). We found that at the cellular level *Per2* is involved in the response of a cell to oxidative stress.

## Experimental Procedures

### Isolation of mouse embryonic fibroblasts

Mouse embryonic fibroblasts (MEFs) from wild type and *Per2^Brdm1^* mutant mice were obtained at E13.5–14. Embryos were dissected under sterile conditions, the heads and the internal organs were discarded, and the rest of the bodies were minced and passed through a 2 ml bent syringe into a 60 ml bottle. Embryos were incubated in 5 ml trypsin/2 mM EDTA at 37°C – 5% CO_2_ for 15 min, pelleted (4000 g for 5 min), resuspended in 10 ml of fresh DMEM + 10% FCS, and plated in 60 mm dishes (1:5 dilution). The next day, the medium was removed the plates were rinsed twice with TBS and covered with 10 ml of fresh medium. The plates were then incubated for 24 h at 37°C – 5% CO_2_ and split 1:10. About 90% confluent MEF’s were frozen in FCS + 10% DMSO and stored in liquid nitrogen until used. At least three cell lines for every genotype were obtained and tested for paraquat sensitivity.

### Enzymatic assays

Cells (passage 4–5) were cultured in DMEM/10% FCS until confluency was reached, then trypsinized and collected for the enzymatic assays (ca 6 × 10^6^ cells). The aconitase assay was performed according to Gardner et al. (14). The SOD assay was done according to the manufacturers instruction in the Trevigen kit (order # 7500-100-K). Lactic dehydrogenase (LDH) assay was performed according to Gardner et al. (15). Cell homogenates were prepared in ice-cold PBS pH 7.4 as described above. The assay was performed in 200 μl reaction mixture containing 50 mM PBS, pH 7.4, 50 μl of sample, 0.2 mM NADH, and 1 mM sodium pyruvate. The decrease in absorbance at 340 nm was recorded for 3 min at 25°C and correlated to LDH activity using a standard curve. LDH activity was normalized for the protein amount.

Total NADH/NAD^+^ ratio in cell extracts was estimated by the lactate/pyruvate ratio (16, 17). Lactate and pyruvate were measured in whole cell extracts (ca 6 × 10^6^ cells) of wild type and *Per2^Brdm1^* mutant cells synchronized by a 100 nM dexamethasone shock (see last paragraph) and taken 6 h after the shock. The samples were tested for lactate and pyruvate levels by adding to the reaction mixture (glycine buffer pH 10.0, 25 μl of sample and 10 units of lactate dehydrogenase) 0.2 mg of NAD^+^ or NADH, respectively. The increase or the decrease in absorbance at 340 nm was recorded after 15 min and correlated to lactate and pyruvate concentration through standard curves. Values were calculated using the formula in Ref. (16) with *K*_eq_ = 4.4 × 10^−2^. Complex I activity was measured according to Chretien et al. and Klement et al. (18, 19).

### Paraquat, plumbagin, hydrogen peroxide, SIN-1, and UV light treatments

Mouse embryonic fibroblasts were treated with paraquat (Supelco, PS-366), plumbagin (Sigma P7262), SIN-1 (Sigma M-5793), and hydrogen peroxide (Sigma H-1009) as reported in Ref. (20) or treated with ultra-violet (UV) light. Briefly, cells were plated in DMEM-10%FCS until confluent and then seeded in 96-wells plates (3000–5000 cells/well). The day after the medium was replaced and paraquat and H_2_O_2_ were added at different concentrations (0, 200, 400, 600, 800, 1000 μM, total volume 150 μl). Plumbagin was added at a final concentration of 1.5 μM, SIN-1 at a concentration of 2 mM, or cells were exposed to UV light at 120 mJ/cm^2^. After 24 h, the cytotoxic effect was measured by a commercial kit based on the colorimetric determination of the LDH released upon cell lysis (Promega, ≠ G-1780). The cytotoxicity was evaluated by calculating the ratio between the LDH released spontaneously and the total LDH contained in the cells and expressed as percentage of cytotoxicity (dead cells relative to the total number of cells). The rescuing of paraquat cytotoxicity through *N*-acetyl-cysteine (NAC) was performed according to Macip et al. (21) using 50 μM NAC.

### Crystal violet staining

Cells were seeded into 24 well plates (4 × 10^4^ cells/well) and treated with paraquat, NAC, or paraquat + NAC for 24 h (see above). Medium was aspired; cells were washed once with PBS and incubated with 0.02% crystal violet/2% EtOH for 20 min at room temperature. Plates were thoroughly rinsed with tap water and dried. Crystal violet was solubilized in 1% SDS, and sample absorption was read at 595 nm.

### Construction of a recombinant adenovirus expressing the full length Per2 ORF (*Ad-Per2*)

The *Per2* gene, cloned into a Tet-repressible expression vector (pSCOT), was inserted into the E1 region of a cloned ΔE1ΔE3 adenoviral backbone (vmRL-CMV1) through homologous recombination in *E. coli* BJ5183. Bacteria were transformed by electroporation (2.5 kV in 0.2 cm gap cuvettes, 25 μF, 200 Ω) with the vector carrying the Per2 gene and the virus mid. Both constructs were carrying the CMV promoter and a region spanning the rabbit β-globin intron 2 and the β-globin exon 3, where the homologous recombination occurred. Positive clones were screened by PCR and the virus mids were isolated from the bacteria and purified by a standard equilibrium centrifugation in CsCl–ethidium bromide gradient *(22)*. Virus mids were transfected with the calcium–phosphate method in a packaging cell line (HER911 Tet) to get the recombinant adenovirus (F∅ generation). The transfected cells were harvested, lysed by repeated freezing-thawing and centrifuged. The supernatant, containing the viral particles, was used to infect HER911 Tet cells for a larger scale preparation (F1 generation). The titer (TCID_50_) of the F1 viral generation was finally determined with the Reed–Muench method (23).

### Infection of MEFs

Mouse embryonic fibroblasts were seeded in 96-wells plates (3000 cells/well) and infected with the *Ad-Per2* at 600 or 60 m.o.i (100 μl volume of infection, 48 h incubation). Expression of the PER2 protein in cells was confirmed by Western blotting. As a control, MEFs were infected with an adenovirus containing the green fluorescent protein (GFP) gene in order to follow infection by microscopic inspection and to check the effects of the adenovirus on cell viability. After incubation, cells were treated with 600 μM paraquat and incubated for 24 h at 37°C – 5% CO_2_. Cell death was evaluated with the cytotoxicity test mentioned above (Promega). All the experiments were performed in duplicate with populations of wild type or *Per2^Brdm1^* mutant cells composed of three different cell lines equally represented. Data were compared by one-way ANOVA and Bonferroni’s *post hoc* test.

### Circadian profile of sensitivity to paraquat

Cells were grown in 20% FCS to confluency and 100 nM dexametasone was added for clock synchronization (24). Dexamethasone was carefully washed away after 15 min and replaced with fresh DMEM with 20% FCS. Six, 12, 18, and 24 h after dexamethasone shock 600 μM paraquat was added to the wells and after 24 h incubation the cytotoxicity was evaluated as described above.

### H_2_DCFDA staining

Cells were grown in 96-well plates to 80–90% confluency. After washes 25 μM H_2_DCFDA (2′,7′-dichlorodihydrofluorescein diacetate) diluted in phenol-red free medium without FCS were added, and cells were incubated for 30 min at 37°C. Subsequently, cells were washed once with PBS and fresh PBS was added. Fluorescence of DCF (2′,7′-dichlorofluorescein) was measured at 485 nm excitation and 528 nm emission wavelength in a Synergy HT multi-mode plate reader (BioTek). Values for cells treated with phenol-red free medium alone were subtracted from all other values.

### Mitochondrial staining

Wild type and *Per2^Brdm1^* mutant MEFs were cultured in 8-well slide chambers at a density of 10^4^ cells/well. Culturing medium was replaced with fresh medium containing 100 nM MitoTraker^®^Green FM (Molecular Probes, M7514). After 45 min at 37°C, the medium was removed and the slides inspected under the microscope and photographed.

### Semiquantitative PCR

PCR amplification of a 2.3 kb fragment of mitochondrial DNA (mtDNA) was performed by using the primers described in Ref. (25). The PCR was performed as follows: denaturation step of 3 min at 94°C, then 15–35 cycles of denaturation–annealing–extension (denaturation 94°C 1 min, annealing 55°C 2 min, extension 72°C 2 min). After a final extension step of 10 min at 72°C, the tubes were chilled on ice and 8 μl of PCR products were loaded onto a 1% agarose gel.

For analysis of the circadian expression profile of *Cry1* in synchronized cultures, cells were synchronized with dexamethasone as described above and samples were prepared 0, 3, 6, 12, 18, and 24 h after synchronization. For the assessment of *bcl-2* expression following paraquat treatment, cells were treated with paraquat for 24 h. Total RNA was prepared using RNAzolB (WAK Chemie Steinbach, Germany; WAK-CS-1005) according to manufacturer’s instructions. Contaminating genomic DNA was removed using DNA-free (Ambion, 1906) according to manufacturer’s instructions followed by phenol:chloroform extraction. RNA integrity was checked on a 0.8% agarose gel and concentration was determined spectrophotometrically. Two micrograms of total RNA were reverse transcribed using SuperScriptII reverse transcriptase (Invitrogen, 18064-014).

For amplification of *histone2Az*, *bcl-2*, and *Cry1*, the following primers were used:

*H2Az* forward 5′-CGTATTCATCGACACCTGAAA-3′
*H2Az* reverse 5′-CTGTTGTCCTTTCTTCCCGAT-3′
*bcl-2* forward 5′-CCCCACCGAACTCAAAGAAG-3′
*bcl-2* reverse 5′-CGGGAGAACAGGGTATGATA-3′
*Cry1* forward 5′-CCTGGACAAGATCATAGAACTCA-3′
*Cry1* reverse 5′-CCAAAGCGGAGATAAGGACTGAG-3′

PCR conditions were 30 s denaturation at 94°C, 30 s primer annealing (*H2Az*: 50°C, *bcl-2*: 56°C, *Cry1*: 54°C), and 1 min elongation for 30–37 cycles. PCR products were run on 1.5% agarose gels, bands were quantified (QuantityOne 3.0, Biorad), and *bcl-2* and *Cry1* expression was normalized to *H2Az*. Subsequently, the data were analyzed by a *t*-test.

### Annexin V and propidium iodide staining

Annexin V and propidium iodide staining were performed using a BD Biosciences kit (no. 550911 and 556463). Cells were plated in an eight chambered slide (5–10 × 10^3^ cells/well) and treated with 600 μM paraquat. Ten and 16 h after the beginning of the treatment the medium was removed and cells were washed once with PBS and annexin V binding buffer (250 μl/well). Cells were then incubated with annexinV-FITC antibody (diluted 1:10 in annexin V binding buffer, 250 μl/well) and propidium iodide (0.5 μg/250 μl) at 37°C in darkness for 30 min.

### GEArray

Apoptosis GEArray Q series membranes (SuperArray Biosciences, Bethesda, MD, USA, No. MM-002) were hybridized with labeled cDNA obtained from mRNA extracted from MEFs of the two genotypes according to the manufacturer’s instructions. The dried array membranes were then scanned using an Odyssey infrared imaging system (LI-COR Biosciences). Images were analyzed using the web-based GEArray expression analysis suite (Super Array Biosciences, Bethesda, MD, USA).

### Western blots

Proteins were separated by SDS-PAGE and transferred onto nitrocellulose membranes. Membranes were incubated with anti-PER2 antibody (Beckton-Dickinson, GP81620-050) 1:1000, anti-p53 antibody (Oncogene, OP03) 1:1000, or anti-actin antibody (Sigma, A5060) 1:1000 (all diluted in blocking buffer) at 4°C over night. They were washed and subsequently incubated with appropriate HRP-conjugated secondary antibodies (anti-rabbit for actin, anti-mouse for PER2 and p53; both Sigma, A9169 and A9044) for 1 h at RT. Detection was performed using the Western blotting detection reagents kit (Amersham Biosciences, 1059243) according to manufacturer’s instructions. Membranes were exposed on Hyperfilm (Amersham Biosciences, RPN1678K). For quantitative analysis, bands were quantified (QuantityOne 3.0, Biorad), and band intensity was normalized to actin.

## Results

### Elevated resistance of *Per2^Brdm1^* mutant cells to oxidative stress

**Figure 1:**
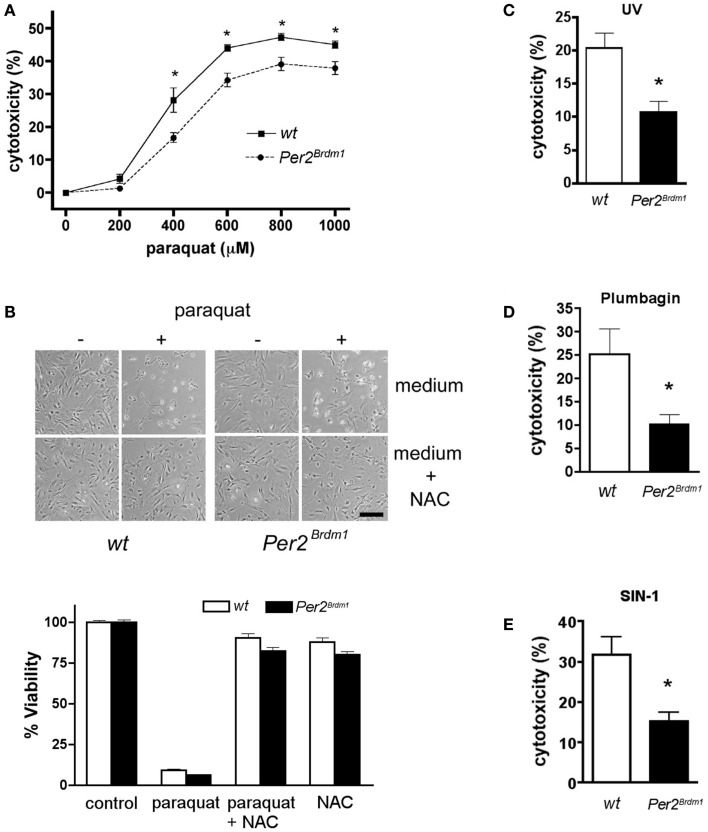
**Mutation in the clock gene *Per2* leads to altered response of asynchronous cells to oxidative stress**. **(A)** Mouse embryonic fibroblasts (MEFs) of wild type (*wt*, solid line) and *Per2* mutant mice (*Per2^Brdm1^*, hatched line) display differences in cytotoxicity in response to various amounts of paraquat. LDH released into the medium versus LDH in living cells was measured after 24 h of paraquat treatment to determine cytotoxicity (*n* = 4–5 MEF preparations per genotype, *p < 0.05 two-way-ANOVA with subsequent Bonferroni test). Cell death due to plating was deducted (*wt* = 7 ± 0.8%, *Per2^Brdm1^* = 7.4 ± 1%, *n* = 3) **(B)** *N*-acetyl cystein (NAC), a radical scavenger, reverses paraquat mediated cytotoxicity. About 100 mM NAC was added to MEFs and subsequently the cells were treated with 600 μM paraquat. Top: photomicrographs of cells. Scale bar = 200 μm. Bottom: quantification of viability using the crystal violet method. **(C)** Wild type (*wt*, white bar) and *Per2* mutant (*Per2^Brdm1^*, black bar) MEFs display differences in cytotoxicity in response to ultra-violet (UV) treatment (120 mJ/cm^2^), in response to plumbagin (1.5 μM) **(D)**, and in response to SIN-1 (2 mM) **(E)** (*p < 0.05).

To evaluate the relationship between *Per2* and the cellular response to oxidative stress, we treated MEFs with paraquat, a chemical that increases ROS production inside the cell by interfering with the oxidative chain by reducing oxygen to oxygen radicals. Comparison between wild type and *Per2^Brdm1^* mutant MEFs revealed a sigmoidal dose response curve to paraquat and a better survival rate for *Per2^Brdm1^* mutant cells after 400–800 μM paraquat treatment (Figure 1A). Application of NAC, a radical scavenger, abolished the cytotoxic effect of paraquat on the cells, indicating that the observed cytotoxicity is due to radicals (Figure 1B). Other ROS generating agents such as UV, plumbagin (26), and SIN-1 treatment confirmed the elevated resistance of *Per2^Brdm1^* mutant cells to oxidative stress (Figures 1C–E). Therefore, we suspected that *Per2* plays a role in the regulation of cellular responses to oxidative stress.

### Rescue of *Per2* mutant MEFs

Expression of *Per2* in *Per2^Brdm1^* mutant cells should rescue the observed phenotype and make these MEFs more susceptible to oxidative stress, comparable to wild-type cells. We introduced the wild-type *Per2* gene into *Per2^Brdm1^* mutant MEFs using an adenovirus to express the PER2 protein under the cytomegalovirus (CMV) promoter in those cells (*Ad-Per2*) (see Figure S1 in Supplementary Material). As a control, the same viral vector expressing GFP was used (*Ad-GFP*). After 48 h, we treated the cells with 600 μM paraquat and measured cell death 24 h thereafter. At a multiplicity of infection (moi) of 600 *Ad-Per2* restored paraquat induced cytotoxicity in *Per2^Brdm1^* mutant cells from 37.7 ± 2 (% cytotoxicity ± SEM) to 45.5 ± 3.1%, a level comparable to wild-type cells (43.9 ± 1.3%) (Table 1). The same amount of *Ad-GFP* had no effect (35.2 ± 4.4%). Expression of *Ad-Per2* and *Ad-GFP* in wild-type cells did not alter their response to paraquat significantly (46.6 ± 1.2 and 44.5 ± 1.6%, respectively, Table 1). These results indicate a causal relationship between *Per2* and the modulation of cell death in response to oxidative stress.

**Table 1:** ***Per2* influences sensitivity to reactive oxygen species (ROS)**.

Conditions				Cytotoxicity (%)	
Vehicle	Paraquat	*Ad-Per2*	*Ad-GFP*	*wt*	*Per2^Brdm1^*
+	−	−	−	0.2 ± 0.6	0 ± 1.0
+	+	−	−	43.9 ± 1.3	37.7 ± 2.0*
−	−	+	−	4.3 ± 1.3	4.4 ± 0.6
−	−	−	+	1.4 ± 0.4	0.1 ± 0.9
+	+	+	−	46.6 ± 1.2	45.5 ± 3.1
+	+	−	+	44.5 ± 1.6	35.2 ± 4.4*

MEFs were treated with 600 μm paraquat and infected with 600 moi (moi, multiplicity of infection) of adenovirus containing either the wild-type *Per2* gene (*Ad-Per2*) or green fluorescent protein (*Ad-GFP*). After 24 h, the supernatants of the cultures were assayed for LDH enzyme activity. Cytotoxicity was determined by the ratio of released LDH over maximal LDH. Cell death due to plating was deducted (*wt* = 7.8 ± 0.4%, *Per2^Brdm1^* = 7.8 ± 0.5%, *n* = 3). The data are presented as mean ± SEM of three experiments. *p < 0.05.

### Time dependence in paraquat sensitivity and oxidative state

**Figure 2:**
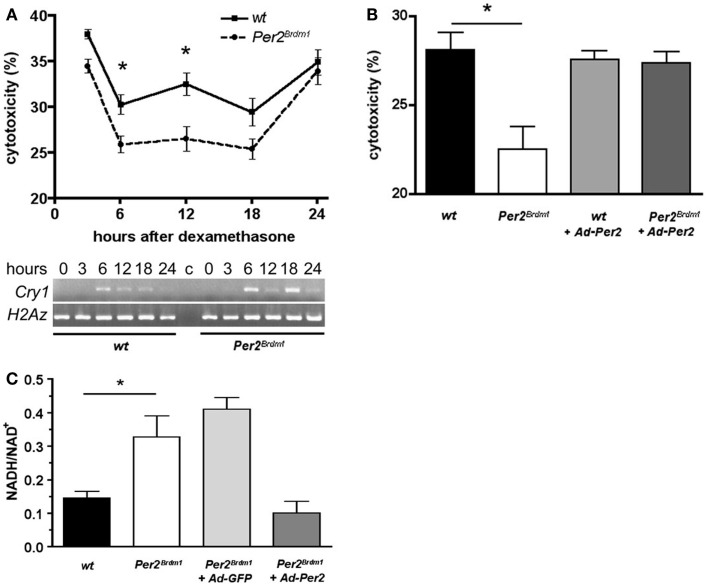
**Time dependent sensitivity of synchronized wild type and *Per2^Brdm1^* mutant MEFs to paraquat and altered oxidative state in *Per2^Brdm1^* mutant MEFs**. **(A)** Cells were synchronized with dexamethasone and treated with paraquat after the indicated times. LDH released into the medium versus LDH in living cells was measured after 24 h of paraquat treatment to determine cytotoxicity. Cytotoxicity of paraquat in both genotypes is time dependent and significantly reduced in *Per2^Brdm1^* mutant MEFs (*p  < 0.05, *n* = 3). The panel below shows clock synchronization of MEFs monitored by expression of *Cry1* relative to the histone H2Az. Wild-type cells show a significant 24 h cycling (*p  < 0.05, *n* = 3) whereas *Per2^Brdm1^* mutant MEFs display altered *Cry1* expression. Significant differences in *Cry1* expression in these cells at 6 and 18 h are observed (*p  < 0.05, *n* = 3). **(B)** Rescue of sensitivity toward paraquat in *Per2^Brdm1^* mutant MEFs 6 h after dexamethasone treatment by introduction of the wild-type *Per2* gene into *Per2^Brdm1^* mutant MEFs using adenovirus (*Ad-Per2*) (*p < 0.05, *n* = 3). **(C)** NADH/NAD^+^ ratio is elevated in *Per2^Brdm1^* mutant MEFs (*p < 0.05, *n* = 3). This ratio is normalized to wild-type levels by introduction of the wild-type *Per2* gene (*Ad-Per2*) but not *GFP* (*Ad-GFP*) using adenovirus (*n* = 3).

As a next step, we addressed the question whether the sensitivity of the cells to paraquat is of circadian nature. Therefore, we synchronized the circadian clocks in MEFs using dexamethasone as described previously (24) and monitored expression of the clock gene *Cry1* to confirm synchronization of the cellular clocks (Figure 2A, bottom panel). After dexamethasone application and subsequent 24 h paraquat treatment maximal cytotoxicity is observed in wild type and *Per2^Brdm1^* mutant MEFs 3 and 24 h after clock synchronization. However, at times in between (6, 12, and 18 h) cytotoxicity is decreased (*p* < 0.0001) (Figure 2A). Consistent with the finding in non-clock-synchronized cells (Figure 1A), significant differences in sensitivity to paraquat between wild type and *Per2^Brdm1^* mutant MEFs can be observed (Figure 2A, 6 h after dexamethasone: wild type: 30.3 ± 1%, *Per2^Brdm1^*: 25.9 ± 0.9%; cytotoxicity in% ± SEM, *p * < 0.05, and 12 h after dexamethasone: wild type: 32.5 ± 1.2%, *Per2^Brdm1^*: 26.5 ± 1.3%; cytotoxicity in% ± SEM, *p * < 0.01). It appears that clock synchronized MEFs cultured in 20% serum are in general more resistant to paraquat compared to asynchronous cells grown in presence of 10% serum. The reason for this difference is that serum contains radical scavengers and hence cultures with higher serum content have a higher capacity to buffer ROS. Introduction of the wild-type *Per2* gene into *Per2^Brdm1^* mutant MEFs 6 h after dexamethasone treatment results in an increase of sensitivity to the cytotoxic effects of paraquat and reaches a level observed in wild-type MEFs (Figure 2B, wild type: 28.1 ± 1%, *Per2^Brdm1^*: 22.5 ± 1.3%, wild type + *Ad-Per2*: 27.6 ± 0.5%, *Per2^Brdm1^* + *Ad-Per2*: 27.4 ± 0.6%; cytotoxicity in% ± SEM, *p* < 0.05). This is accompanied by an alteration in the oxidative state (Figure 2C). We find that the NADH/NAD^+^ ratio is significantly higher in clock synchronized *Per2^Brdm1^* mutant cells compared to wild type (wild type: 0.14 ± 0.02, *Per2^Brdm1^*: 0.33 ± 0.06, NADH/NAD^+^ ± SEM, *p* < 0.05). This ratio can be normalized to wild-type levels when the complete *Per2* gene is introduced via adenovirus into *Per2^Brdm1^* mutant MEFs (0.1 ± 0.04), but not when GFP is introduced (0.41 ± 0.03, Figure 2C). Over expressing the *Per2* gene in *Per2^Brdm1^* mutant MEFs probably has a strong influence on the cellular clock. Therefore, it is not clear whether the rescue of oxidative state is a direct consequence of *Per2* or whether other clock components such as cryptochromes are the mediators of this process. These results, however, indicate that *Per2* is involved in regulating the oxidative state of a cell and influences the cellular response to oxidative stress in a direct or indirect manner.

### Endogenous ROS, sensitivity to peroxide and mitochondrial function

**Figure 3:**
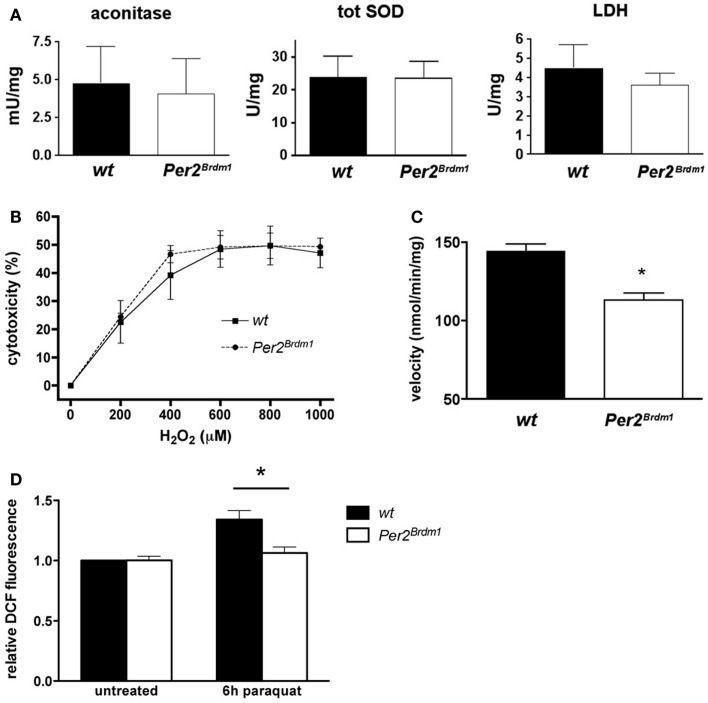
**Endogenous reactive oxygen species (ROS) and sensitivity to H_2_O_2_ are similar in wild type and *Per2^Brdm1^* mutant MEFs but mitochondrial function is different between the two genotypes**. **(A)** Enzymatic activities of aconitase, total superoxide dismutase (SOD), and lactate dehydrogenase (LDH) in wild type and *Per2^Brdm1^* mutant MEFs reveal no differences in the amounts of endogenous ROS between the two genotypes (*n* = 3). **(B)** The sensitivity to H_2_O_2_ is comparable between wild type and *Per2^Brdm1^* mutant MEFs pointing to similar cell membrane sensitivity of the two genotypes to this compound (*n* = 3). Cell death due to plating was deducted (*wt* = 9.2 ± 2%, *Per2^Brdm1^* = 7.4 ± 2.4%, *n* = 3) **(C)** Activity of complex I (NADH ubiquinone oxidoreductase) of the oxidative chain is significantly reduced in *Per2^Brdm1^* mutant compared to wild-type MEFs suggesting altered mitochondrial function in *Per2^Brdm1^* mutant MEFs (*p < 0.05, *n* = 5). **(D)** Assessment of intracellular ROS levels after 6 h of paraquat treatment using H_2_DCFDA. ROS mediated conversion to DCF was measured and values were normalized to untreated wild-type MEFs (*p  < 0.05, *n* = 4, unpaired *t*-test).

To find whether the *Per2* gene influences the endogenous amount of ROS in a cell, we evaluated the endogenous amount of ROS present in wild type and *Per2^Brdm1^* mutant MEFs via aconitase activity, an enzyme of the Krebs cycle that is sensitive to ROS (14). Aconitase activity in both wild type and *Per2^Brdm1^* MEFs is comparable and suggests the presence of similar amounts of endogenous ROS in both genotypes (Figure 3A). Because ROS, particularly superoxide anions, can be eliminated in a cell by SOD (27), we measured the amount of total SOD activity (cytoplasmic and mitochondrial) in both genotypes. No differences were observed (Figure 3A), which is consistent with the aconitase assay. There were also no significant differences between the two genotypes in the activity of the control enzyme lactate dehydrogenase (LDH) (Figure 3A). Additionally, LDH did not show a circadian activity pattern in wild type and *Per2^Brdm1^* mutant brain tissue (see Figure S2 in Supplementary Material). These observations indicate that the levels of total endogenous ROS in wild type and *Per2^Brdm1^* mutant MEFs are not significantly different under unstressed conditions. However, sub-cellular differences in ROS production cannot be excluded.

To decipher whether the higher resistance of *Per2^Brdm1^* mutant MEFs toward oxidative stress was due to the intracellular defense system or to a higher strength of cell membranes toward lipid peroxidation, hydrogen peroxide (H_2_O_2_) was applied to the cells. Exposure to high H_2_O_2_ concentrations for long periods induces necrotic rather than apoptotic cell death by direct damage of cell membranes (28). After 24 h exposure to H_2_O_2_ at different concentrations no significant difference between *Per2^Brdm1^* mutant and wild-type MEFs could be observed (Figure 3B) indicating that the resistance of *Per2^Brdm1^* mutant cells toward paraquat, plumbagin, SIN-1, and UV treatment is not the result of ROS scavenging processes at the cell membrane. Therefore, we tested enzymatic activities in mitochondria that contribute to radical production. We measured the activity of complex I (NADH ubiquinone oxidoreductase) of the oxidative chain in the mitochondria, which transports electrons from NADH to ubiquinone and thereby influences the oxidative state of a cell (Figure 3C). We find that its activity under saturating conditions is significantly lower in *Per2^Brdm1^* mutant cells (wild type: 144.4 ± 4.5 nmol/min/mg, *Per2^Brdm1^* mutant: 113.1 ± 4.6 nmol/min/mg, *p* = 0.0012) indicating that production of oxygen radicals through interference of paraquat with complex I is reduced. In order to test this conjecture, we determined intracellular ROS levels after paraquat treatment using H_2_DCFDA (2′,7′-dichlorodihydrofluorescein diacetate), which is oxidized by ROS [especially H_2_O_2_ (29)] to the highly fluorescent DCF (2′,7′-dichlorofluorescein) (30). As hypothesized, the changes in intracellular ROS after 6 h of paraquat treatment were minimal in *Per2^Brdm1^* mutant MEFs, whereas a clear increase was observed in wild-type MEFs (Figure 3D). No changes were seen at basal levels, which is in line with the observation in Figure 1B. These results indicate that the lower sensitivity of *Per2^Brdm1^* mutant MEFs to paraquat is not due to altered basal ROS levels, but to a decrease in ROS production in response to paraquat exposure.

### Amount of mitochondria and *bcl*-2 expression

**Figure 4:**
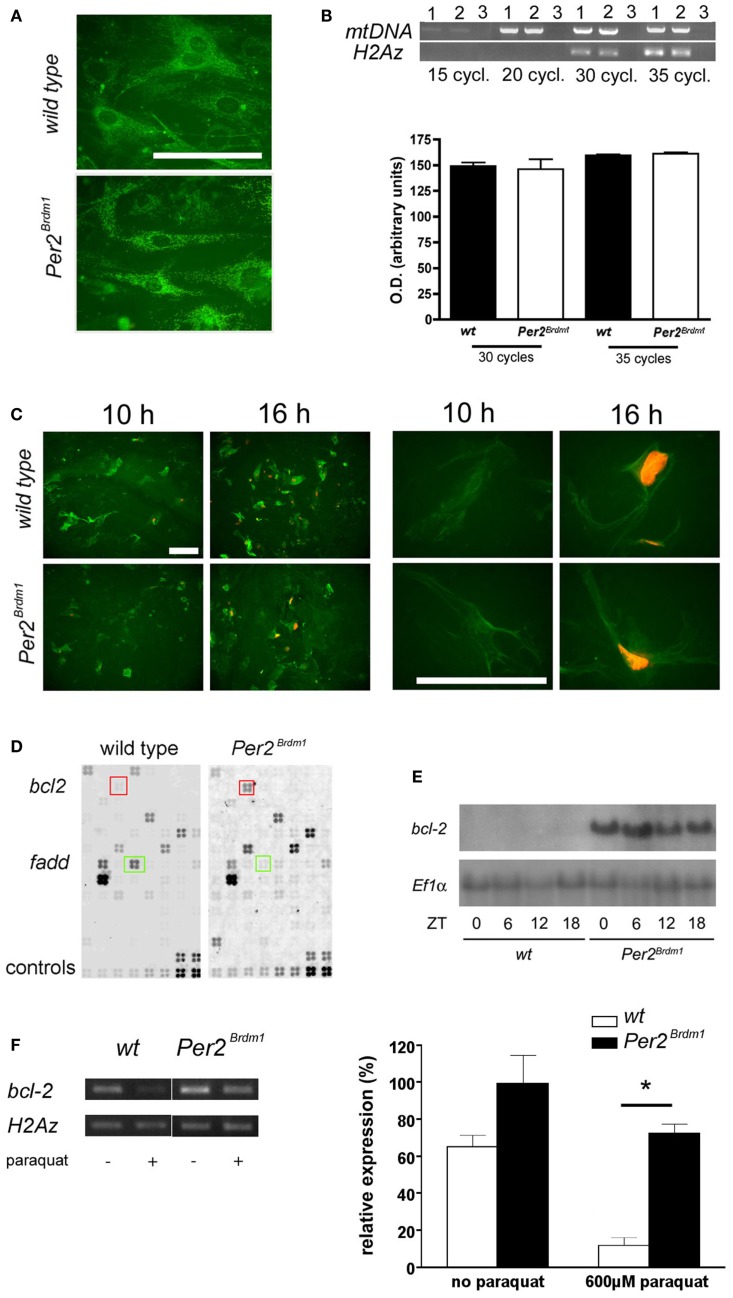
**Amount of mitochondrial DNA in MEFs, apoptosis after paraquat treatment and *bcl-2* expression**. **(A)** Labeling of mitochondria with MitoTraker^®^Green FM. No difference in number of mitochondria can be detected between wild type and *Per2^Brdm1^* mutant MEFs (scale bar = 100 μm). **(B)** Top panel: PCR amplification of a 2.3 kb fragment of mitochondrial DNA (mtDNA) is compared to amplification of a 0.3 kb fragment of nuclear DNA (histone H2Az) after 15, 20, 30, and 35 cycles. Lane 1 = wild type, lane 2 = *Per2^Brdm1^* mutant, lane 3 = no DNA. Similar amounts of mtDNA relative to histone H2Az DNA are observed in both genotypes. Bottom panel: quantification of amplified mtDNA normalized to histone H2Az DNA after 30 and 35 cycles (*n* = 3). **(C)** Staining of wild type and *Per2^Brdm1^* mutant MEFs with annexin V (green) and propidium iodide (orange) 10 and 16 h after paraquat treatment at low (left panel) and high (right panel) magnification. Scale bar = 100 μm. **(D)** Example of apoptosis GEArrays after hybridization with labeled cDNA probes of untreated wild-type and *Per2^Brdm1^* mutant MEFs. Three arrays per genotype were hybridized and genes significantly changed in expression are marked by colored squares, *bcl-2* (red) and *fadd* (green). Controls (two bottom rows) were PUC18 plasmid, blank spots (non-specific binding), *Ppia* and *Rpl13a* as positive controls and two housekeeping genes for normalization (*b-actin* and *Gapdh*). **(E)** Northern blot of mRNA isolated from livers of wild-type and *Per2^Brdm1^* mutant mice sacrificed at the indicated time points. **(F)** Expression of *bcl-2* in wild type and *Per2^Brdm1^* mutant MEFs before and after paraquat treatment (*n* = 3, *p  < 0.05).

Changes in oxidative state have been described for cells depleted of mtDNA (25). Therefore, we tested whether *Per2^Brdm1^* mutant MEFs contain less mitochondria (Figure 4A). Staining with MitoTraker^®^Green, a dye specifically labeling mitochondria regardless of mitochondrial membrane potential, did not reveal gross differences between wild type and *Per2^Brdm1^* mutant cells. In addition, semiquantitative PCR did not uncover differences in mtDNA contents relative to nuclear DNA (Figure 4B). Therefore, we conclude that our findings are not due to less mitochondria in *Per2^Brdm1^* mutant MEFs. It appears that the described observations are the consequence of transcriptional and/or posttranscriptional events regulated by the *Per2* gene. To evaluate whether the MEFs used in the described experiments undergo apoptosis, we took advantage of the fact that apoptotic cells lose plasma membrane asymmetry. We measured the presence of phosphatidylserine (PS), an aminophospholipid that is normally present in the inner leaflet of the plasma membrane, but translocates in early apoptosis to the outer leaflet of the membrane. We measured this process via annexin, a protein with high affinity for PS to indirectly monitor PS translocation. We find that staining for annexin V is already seen 10 h after paraquat treatment (Figure 4C, green color) and only in a few cells DNA was visualized by propidium iodide (Figure 4C, orange color). This indicates that 10 h after paraquat treatment cells are in early apoptosis and the cell membranes are still intact preventing penetration of propidium iodide into the cells. Sixteen hours after paraquat treatment labeled DNA is observed in most cells (Figure 4C, orange color) indicating that cell membranes are damaged typical for late apoptosis. In the cytotoxicity experiments described above assessment was made 24 h after paraquat treatment and hence our evaluation includes cells with defective membranes. To obtain a quantifiable measure for apoptosis, we investigated the expression levels of *bcl-2*, a well-characterized regulator of apoptosis (31). Using an apoptosis pathway cDNA-array, we found that expression of the anti-apoptotic gene *bcl-2* is increased in *Per2^Brdm1^* mutant cells compared to wild-type (Figure 4D) and were able to confirm this observation in liver tissue (Figure 4E). Interestingly, the pro-apoptotic gene *fadd* was down-regulated in *Per2^Brdm1^* mutant cells (Figure 4D). After paraquat treatment, there is a significantly stronger decrease in the expression of *bcl-2* in wild-type cells as compared to *Per2^Brdm1^* mutant cells (Figure 4F). Overall, these data suggest that apoptotic signaling pathways are different between wild-type and *Per2^Brdm1^* mutants supporting our observation that *Per2* modulates cell death in response to oxidative stress.

## Discussion

The present report provides evidence that the *Per2* gene plays a role in the cellular response to oxidative stress and cell death. This might be achieved through the regulatory potential of the *Per2* gene on the redox state of a cell. The redox state of a cell has been proposed to be crucial for the regulation of clock gene dependent transcription via the NAD dependent enzyme SIRT1 (32, 33). Additionally, NAD modulates the DNA binding factors Clock and NPAS2 and their transcription potential (34). NADH favors formation of heterodimers between CLOCK or NPAS2 with BMAL1, which then bind to DNA at E-box promoter elements, thus regulating the efficiency with which many circadian clock and clock controlled genes are transcribed. Therefore, one would expect that the elevated amount of NADH in *Per2^Brdm1^* mutant MEFs would favor the formation of heterodimeric CLOCK/BMAL1 or NPAS2/BMAL1 complexes and lead to an elevated transcription of target genes such as cryptochromes. This might partially compensate for the reduction in expression of *Npas2* and *Bmal1* in *Per2^Brdm1^* mutant cells and explain why *Per2^Brdm1^* mutant mice can display circadian behavior for several days in constant darkness before they lose a circadian activity rhythm (9, 35). This view is compatible with the suggestion that redox regulation is part of the clock mechanism (36, 37). In this model, redox state does not only influence clock protein heterodimerization and thus might contribute to the transcriptional efficiency but redox state might also be regulated by clock components for which evidence is presented in this study. Hence, a feedback loop on redox state might involve the circadian clock (37). It makes sense that the clock and the redox state are interlaced since environmental oxidative stress on a cell has a diurnal pattern (38). Cells of an organism have evolved to meet time dependent oxidative stress and probably use the circadian clock for this purpose (see Figure 2A) (39). A mutation in a clock component, as shown here for *Per2*, will therefore alter the cellular response to oxidative stress in mammalian cells. A change in redox state as manifested by elevated levels of NADH might increase the radical scavenging properties of a cell (40), which leads to better survival in response to oxidative stress. This puts an increased pressure on the DNA repair mechanism to avoid accumulation of mutations. Interestingly, however, p53 protein is reduced in its expression in *Per2^Brdm1^* mutant MEFs [Figure S3 in Supplementary Material, see also Ref. (10)]. This is probably due to the reduced activity of NADH ubiquinone oxidoreductase (Figure 3C), which has been implicated in the regulation of p53 stability and p53-dependent apoptosis (41). Additionally, *Per2^Brdm1^* mutant MEFs do not reduce expression of the anti-apoptotic gene *bcl-2* as strong as wild-type cells in response to the ROS inducing agent paraquat. Therefore, *Per2^Brdm1^* mutant MEFs undergo less apoptosis and survive better under oxidative stress *in vitro*. However, if DNA damage occurs it will lead to tumor formation in an organism *in vivo* as described previously (10, 42). This indicates that the *Per2* gene is not only involved in regulating redox state and response to oxidative stress in a cell but also affects apoptosis via p53 regulation. *Per2* was identified as a component of the p53 pathway in a large-scale RNAi screen in human cells (43) indicating that the relationship between Per2 and p53 is bidirectional (44). Our results based on lack of *Per2* are in agreement with the observation that overexpression of *Per2* in various carcinoma cell lines reduced cellular proliferation with up-regulation of p53 and increased apoptosis that was accompanied by down-regulation of *bcl-2* (45–47). Conversely, flutamide, an anti-prostate cancer drug and apoptosis inducer, up-regulated *Per2* gene expression in prostate mesenchymal cells (48). Interestingly, two cytotoxicity response regulators *Ly49C* and *Nkg2d* are down-regulated in *Per2^Brdm1^* mutant mice (49) supporting a role of *Per2* in the regulation of cytotoxicity responses.

The above discussion illustrates that although a mutation in the *Per2* gene makes cells more resistant to oxidative stress, *Per2^Brdm1^* mutant cells contribute to cancer formation in the organism (10). Hence, the *Per2* gene seems to enhance biological fitness by establishing an optimal balance between cell survival and programed cell death. This does not necessarily lead to maximal longevity under ideal circumstances but optimizes survival of an organism in real life experiencing a variety of different types of environmental stress. In sum, this report provides evidence that the circadian clock gene *Per2* influences cellular response to oxidative stress and modulates cell death, which in turn affects cancer development and the aging process.

## Conflict of Interest Statement

The authors declare that the research was conducted in the absence of any commercial or financial relationships that could be construed as a potential conflict of interest.

## Supplementary Material

The Supplementary Material for this article can be found online at http://www.frontiersin.org/Journal/10.3389/fneur.2014.00289/abstract
