# Supplementary figures and images for: The Mammalian Circadian Clock Gene Per2 Modulates Cell Death in Response to Oxidative Stress
Source: Front Neurol. 2015 Jan 13;5:289. doi: 10.3389/fneur.2014.00289 (PMC4292776; doi:10.3389/fneur.2014.00289)

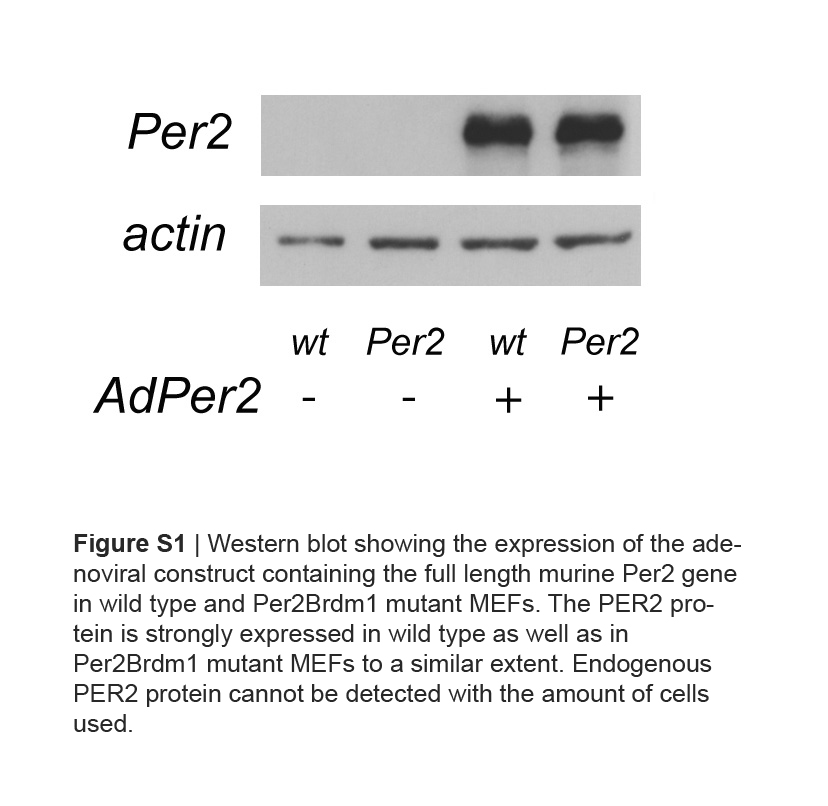

Supplement: Supplementary file 1 [file Image_1.JPEG]

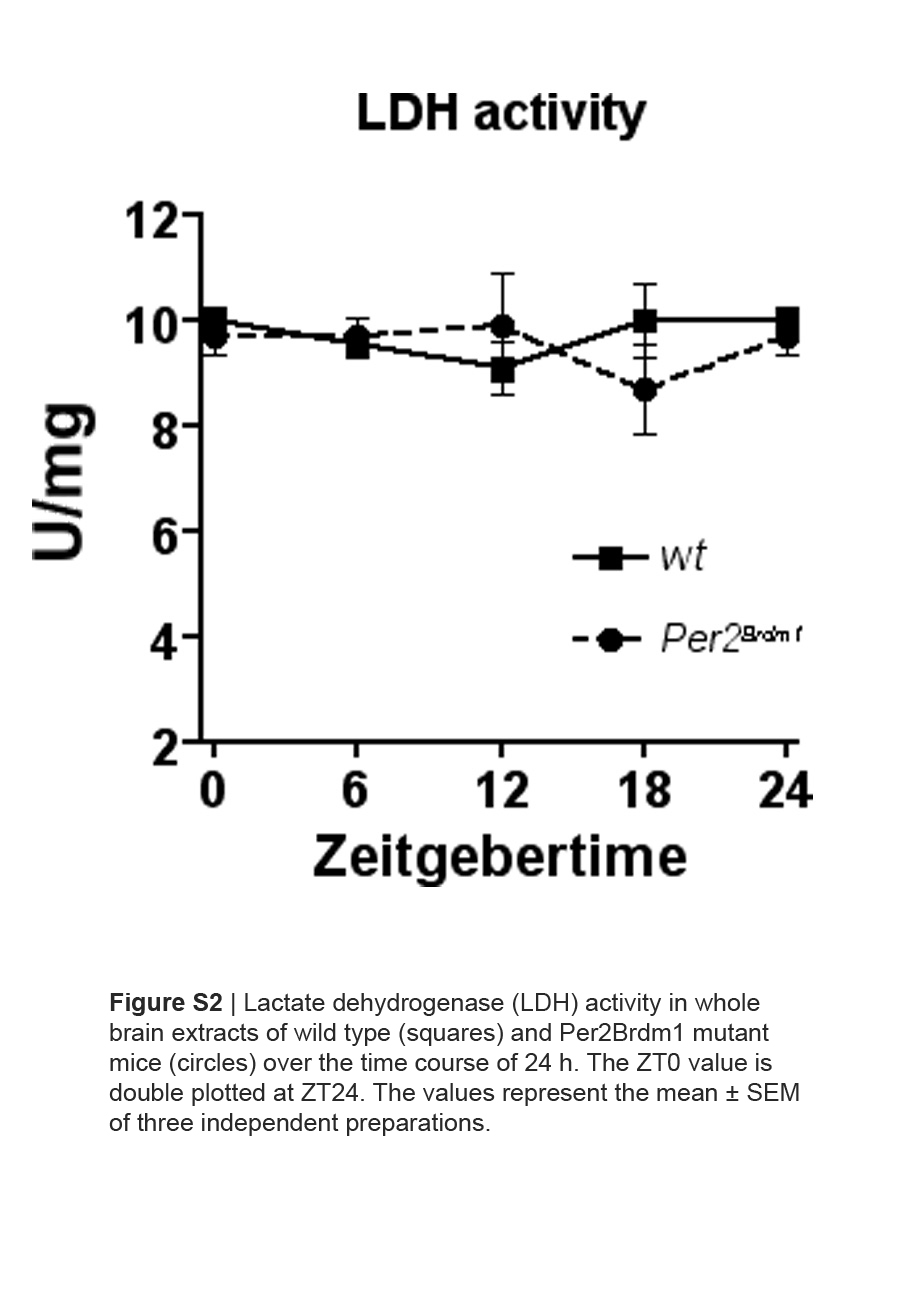

Supplement: Supplementary file 2 [file Image_2.JPEG]

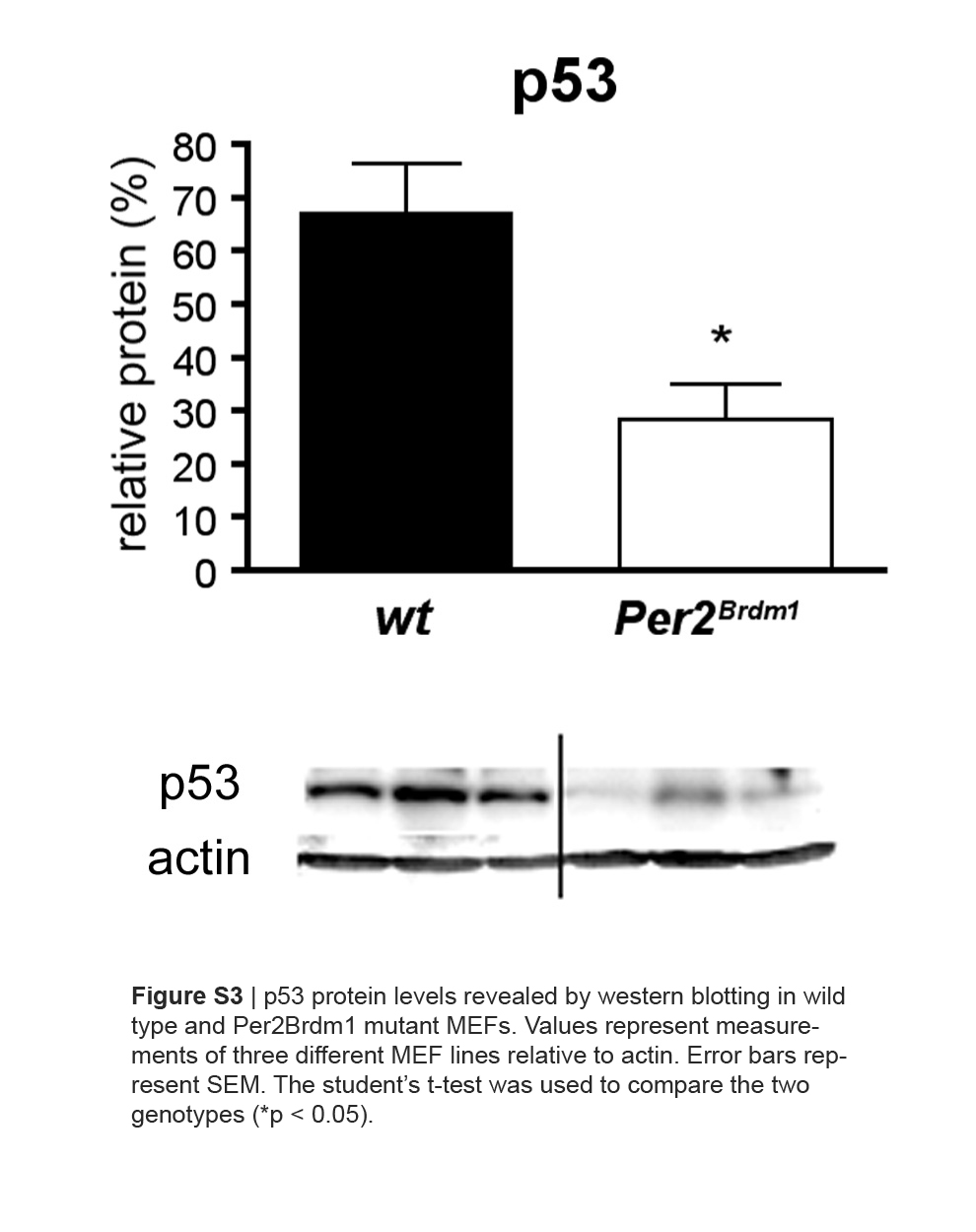

Supplement: Supplementary file 3 [file Image_3.JPEG]
